# Supplementary material for: Single-cell RNA-seq enables comprehensive tumour and immune cell profiling in primary breast cancer
Source: Nat Commun. 2017 May 5;8:15081. doi: 10.1038/ncomms15081 (PMC5424158; doi:10.1038/ncomms15081)
Supplement: Supplementary Information — Supplementary Figures and Supplementary Tables [file ncomms15081-s1.pdf]

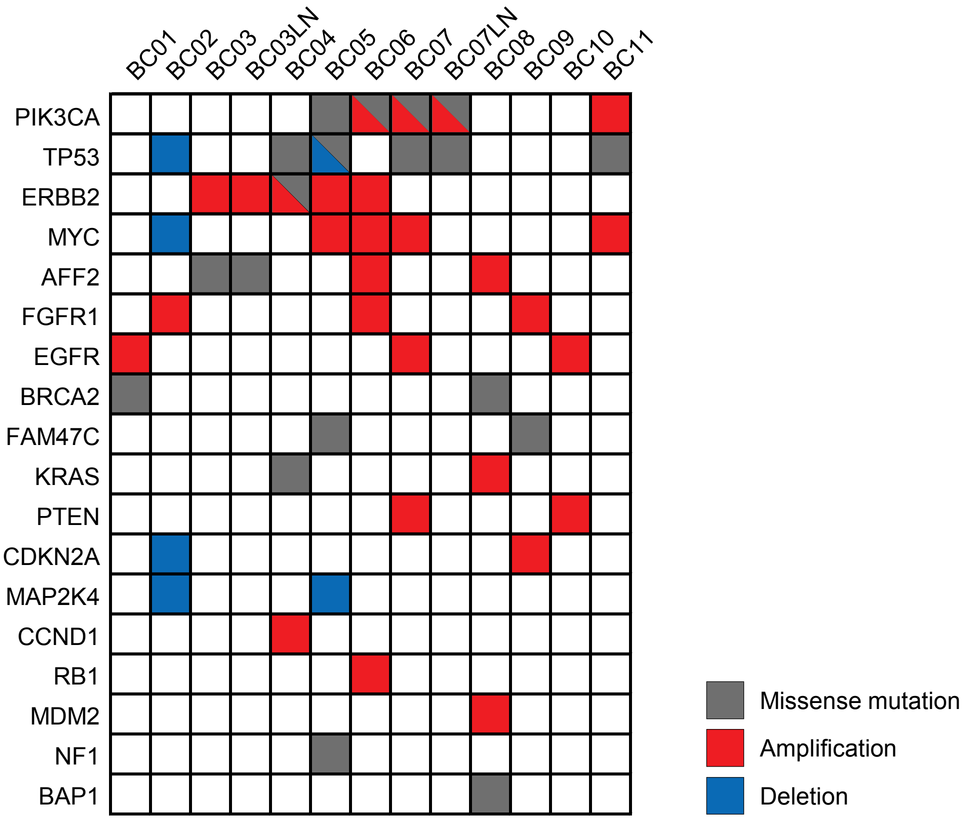

Supplementary Figure 1. Genomic profiles of breast cancer specimens. Copy number variations (CNV) and single nucleotide variations (SNV) were detected from whole exome sequencing data for the bulk tumors. SNV and CNV are marked for significantly mutated genes in breast cancer for each tumor specimen.

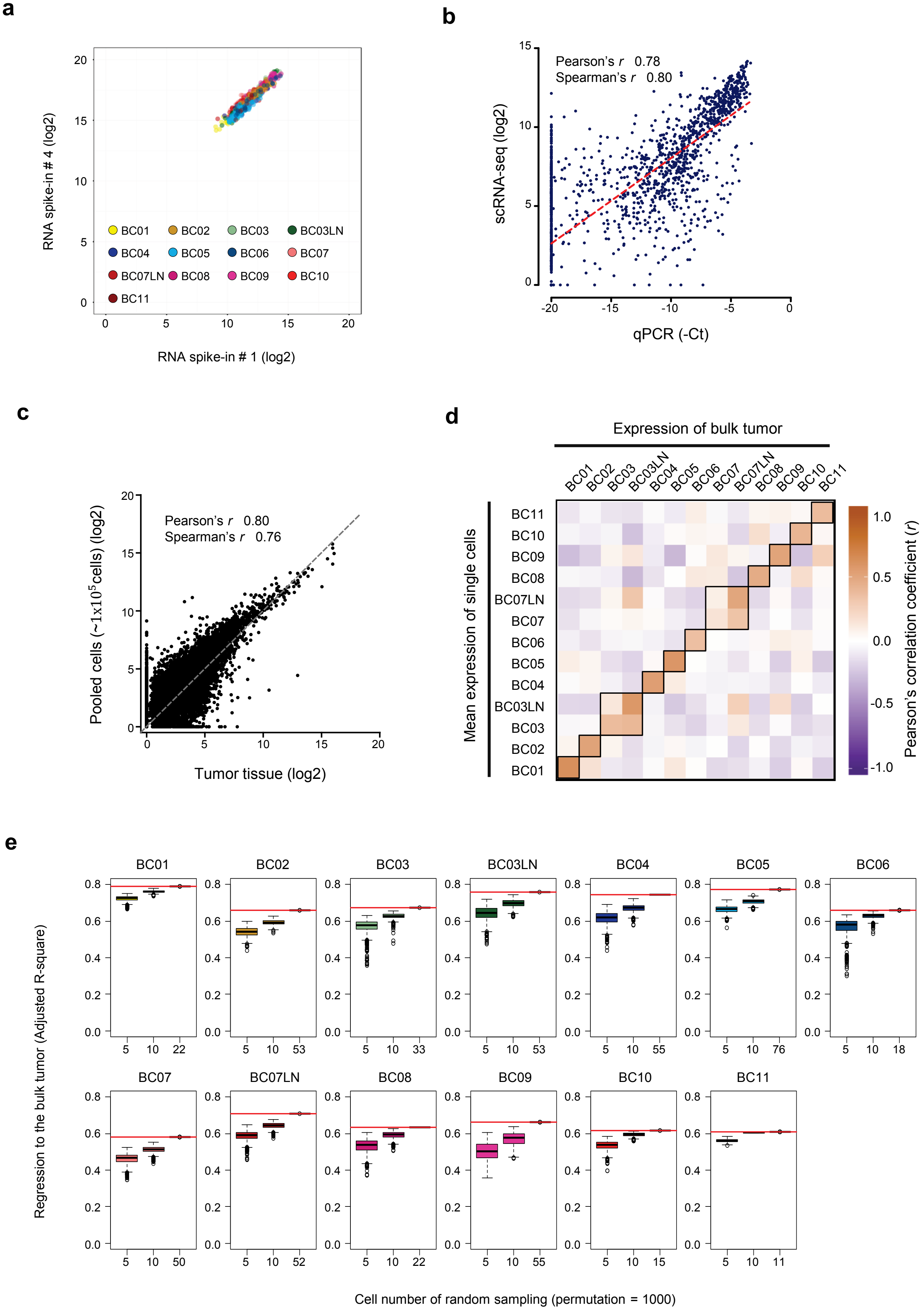

a
RNA spike-in # 4 (log2)
RNA spike-in # 1 (log2)
BC01 BC02 BC03 BC03LN
BC04 BC05 BC06 BC07
BC07LN BC08 BC09 BC10
BC11
b
Pearson's r 0.78
Spearman's r 0.80
scRNA-seq (log2)
qPCR (-Ct)
c
Pearson's r 0.80
Spearman's r 0.76
Pooled cells (~1x10^5cells) (log2)
Tumor tissue (log2)
d
Expression of bulk tumor
Mean expression of single cells
BC01 BC02 BC03 BC03LN BC04 BC05 BC06 BC07 BC07LN BC08 BC09 BC10 BC11
Pearson's correlation coefficient (r)
e
BC01 BC02 BC03 BC03LN BC04 BC05 BC06
BC07 BC07LN BC08 BC09 BC10 BC11
Regression to the bulk tumor (Adjusted R-square)
Cell number of random sampling (permutation = 1000)

Supplementary Figure 2. Reliability and representativeness of single-cell RNA sequencing data. (a) Consistent detection of normalized read counts for two array control RNA spike-ins in all single-cell samples. **(b)** Single-cell RNA-seq data showing a significant correlation with the matched qPCR results (Pearson's *r* 0.78). The linear regression result is drawn as a dashed line. **(c)** Significant correlation in the RNA-seq data between the tumor tissues and pooled tumor tissue isolates (Pearson's *r* 0.8). **(d)** Centered correlations between the averages of tumor single cells and their matching bulk samples (bulk tissue for BC07 or pooled tissue isolates for all others) showing significant but partial representation of the bulk tumor by single cells (Pearson's *r* 0.16-0.63 with average 0.47, $p < 0.001$). **(e)** Multiple regression analysis was performed using expression levels of each single cell as the explanatory variable to predict the expression level of bulk tumors. Adjusted R-squares of multiple regression analysis were calculated by random sampling of single cells with 1,000 iterations. Horizontal red lines represent maximum adjusted R-square values.

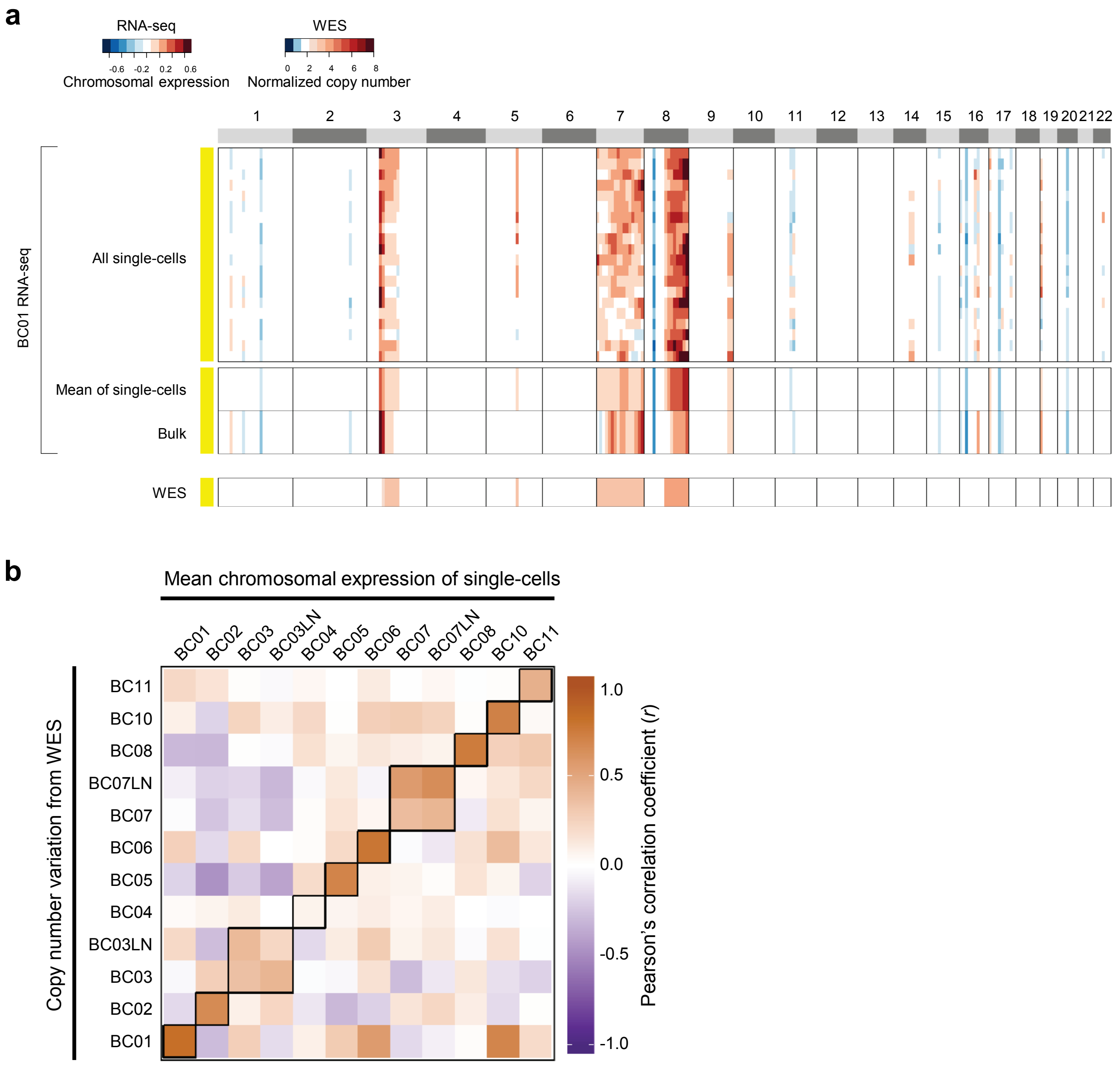

Supplementary Figure 3. The correlation between inferred CNVs from single-cell RNA-seq and CNVs estimated from WES. (a) Heatmaps of estimated CNVs from WES and inferred CNVs from RNA-seq in a 10-Mb genomic window size. In the representative BC01 tumor, the inferred CNVs from RNA-seq has a high correlation with the CNVs from WES (Pearson's *r* 0.82). **(b)** Correlations between averaged inferred CNVs from single-cells and CNVs estimated from WES. Most tumors show a higher correlation with the matched single cell averages than with the unmatched ones (Pearson's *r* 0.08-0.82 with average 0.52).

a

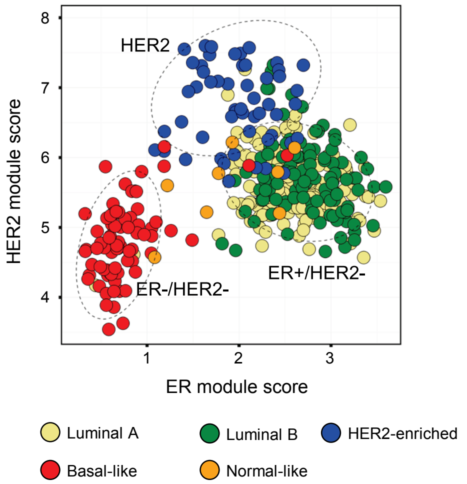

b

Predicted Subtype

TCGA PAM50 Subtype

	ER+/HER2-	HER2+	ER-/HER2-	TOTAL
Luminal A	204	3	2	209
Luminal B	107	5	0	112
HER2-enriched	14	39	0	53
Basal-like	2	1	78	81
Normal-like	6	0	2	8
TOTAL	333	48	82	463
Accuracy	0.93	0.81	0.98	0.91

Supplementary Figure 4. Subtyping validation using TCGA data. (a) Subtyping of TCGA breast cancer samples with the ER and HER2 module scores. **(b)** Subtyping using ER and HER2 module scores predicted the breast cancer subtype for TCGA RNA sequencing data with 91% accuracy.

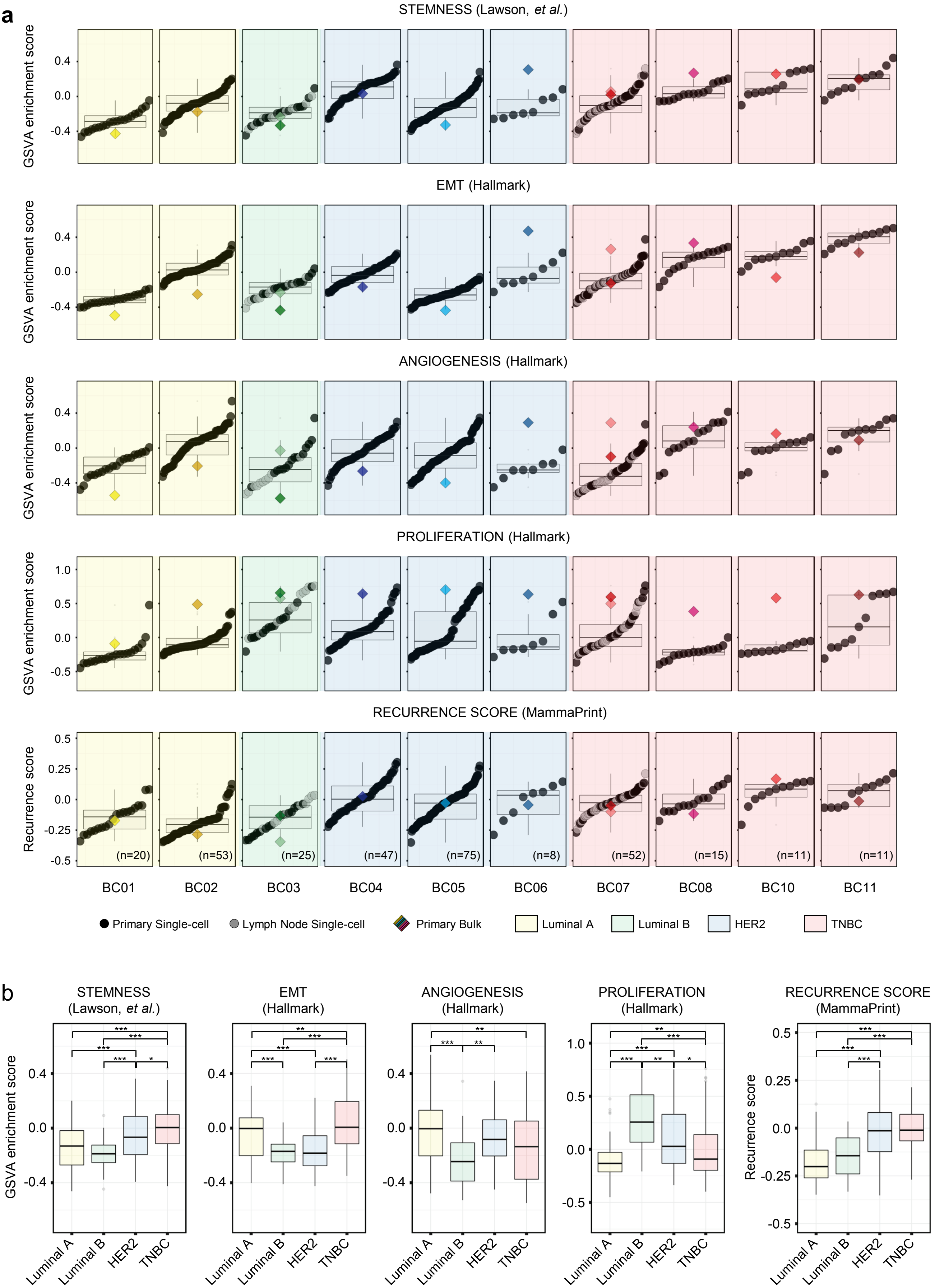
a
STEMNESS (Lawson, et al.)
EMT (Hallmark)
ANGIOGENESIS (Hallmark)
PROLIFERATION (Hallmark)
RECURRENCE SCORE (MammaPrint)
GSVA enrichment score
Recurrence score
(n=20)
(n=53)
(n=25)
(n=47)
(n=75)
(n=8)
(n=52)
(n=15)
(n=11)
(n=11)
BC01
BC02
BC03
BC04
BC05
BC06
BC07
BC08
BC10
BC11
Primary Single-cell
Lymph Node Single-cell
Primary Bulk
Luminal A
Luminal B
HER2
TNBC
b
STEMNESS (Lawson, et al.)
EMT (Hallmark)
ANGIOGENESIS (Hallmark)
PROLIFERATION (Hallmark)
RECURRENCE SCORE (MammaPrint)
GSVA enrichment score
Recurrence score
Luminal A
Luminal B
HER2
TNBC

Supplementary Figure 5. Heterogeneity in cancer-related pathway activation. (a) GSVA enrichment scores in core cancer-related pathways and recurrence scores from MammaPrint[3] (R software package genefu) are plotted for individual tumor cells in four breast cancer subtypes from 10 patient tumors. **(b)** Gene expression signatures in (a) were compared between the four subtypes. Tumor cells from the TNBC subtype showed higher stemness, EMT, and recurrence scores compared to the Luminal subtypes. Each box shows the median and IQR (interquartile range, 25th to 75th percentiles), whiskers indicate the highest and lowest value within 1.5 times the IQR, and outliers are marked as dots. *P* value, Student's *t*-test (***, $p < 0.001$; **, $p < 0.01$; *, $p < 0.05$).

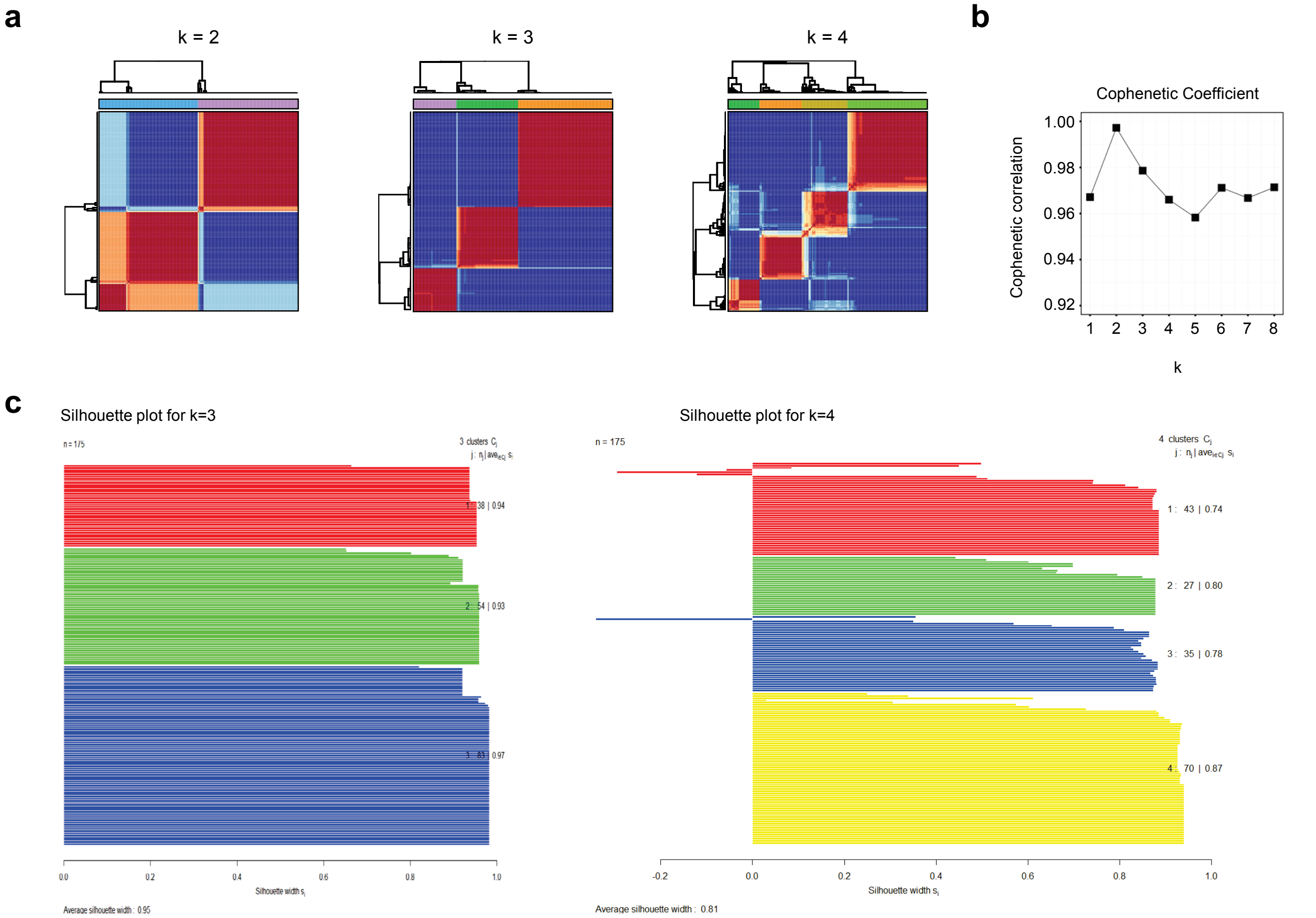

Supplementary Figure 6. NMF clustering for classification of non-tumor cells. (a) NMF clustering analysis with immune gene sets for k = 2, k = 3, and k = 4. **(b)** The curve of cophenetic correlation coefficients. A peak was detected at k = 3, suggesting an optimal cluster of 3. **(c)** The silhouette width of each single cell for the k = 3 and k = 4 clusters. The k = 3 cluster had highest average silhouette width relative to the other clusters.

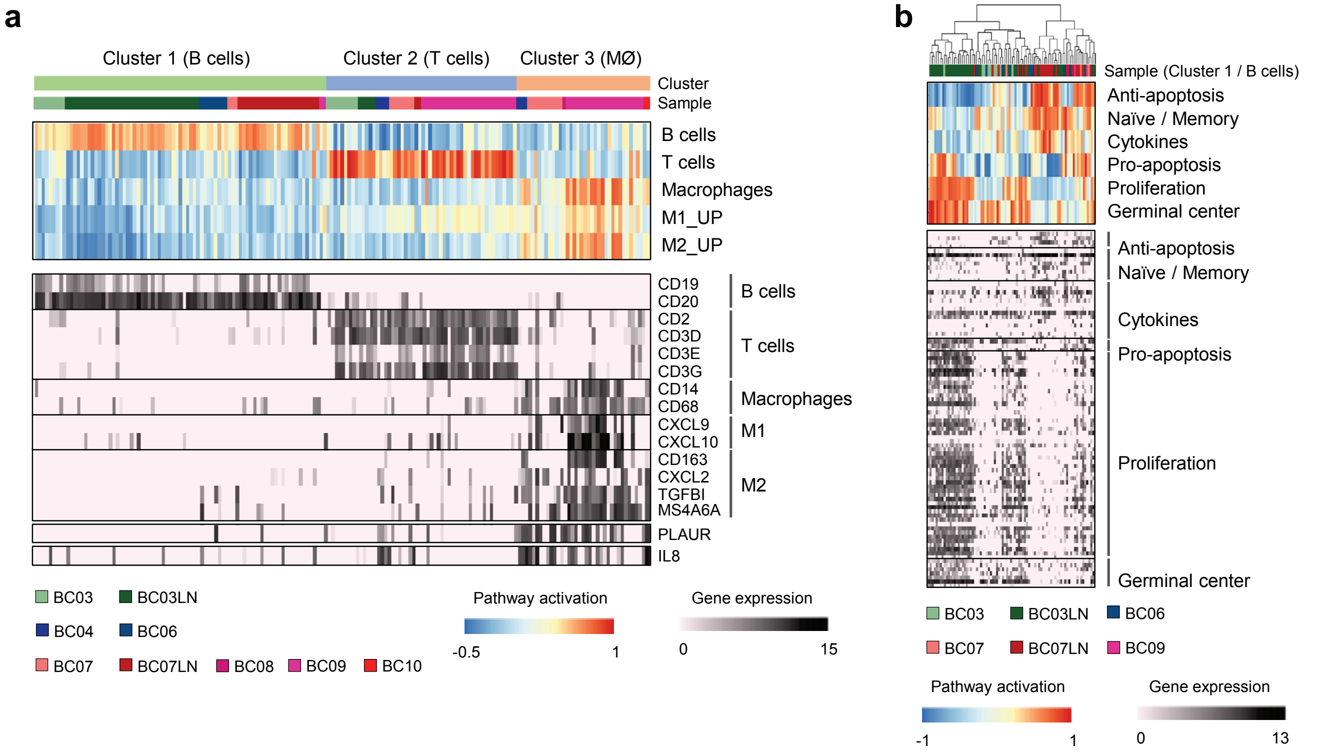

Supplementary Figure 7. Immune signatures in the tumor microenvironment. (a) GSVA analysis using immune genesets (upper panel) shows characteristics of 3 immune clusters. The third cluster, expected as tumor associated macrophages expressed high levels of M2-type genes. **(b)** Hierarchical clustering using GSVA enrichment scores (upper panel) for B lymphocytes[38] classified cells into two groups. One group, mostly from TNBC tumors (BC07, BC07LN, and BC09), showed naïve/memory B cell gene expression signatures. Another group, mostly derived from the BC03 (luminal B tumor) lymph node, showed proliferation signatures associated with germinal center B cells. Gene expression profiles for the used gene sets are presented in the lower panel.

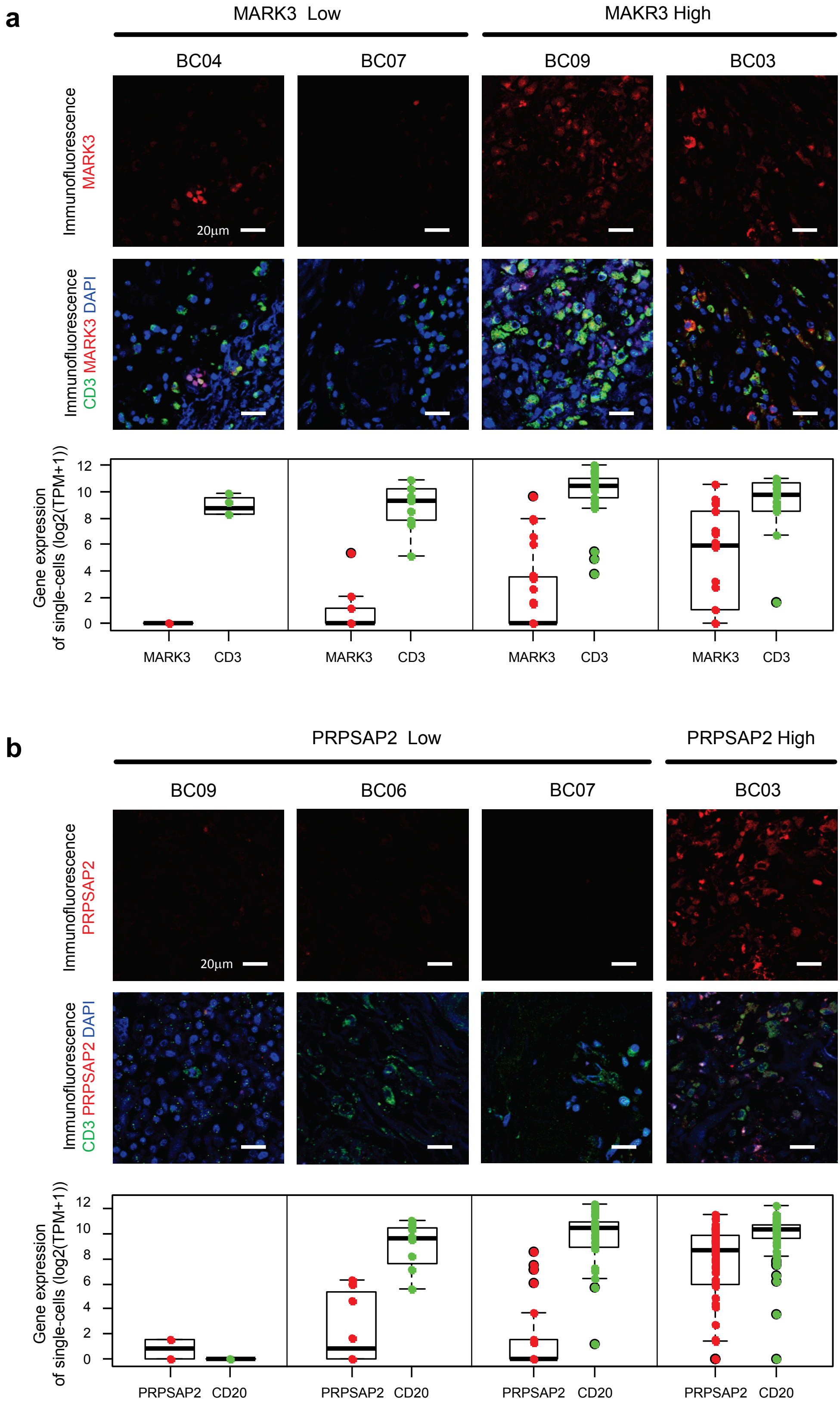

Supplementary Figure 8. Immunostaining with MARK3 and PRPSAP2 in the tumor microenvironment. (a) MARK3 or **(b)** PRPSAP2 were co-stained with the T cell marker CD3 or B cell marker CD20 respectively. Single cell level gene expression is presented at the bottom for comparison. Each box shows the mean and IQR (interquartile range, 25th to 75th percentiles), whiskers indicate the highest and lowest value within 1.5 times the IQR, and outliers are marked as dots. Scale bar, 20μm

Supplementary Table 1. Clinical and histological profiles of the breast cancer specimens

Patient index	BC01 ER+	BC02 ER+	BC03 ER+/HER+	BC04 HER2+	BC05 HER2+	BC06 HER2+	BC07 TNBC	BC08 TNBC	BC09 TNBC	BC10 TNBC	BC11 TNBC
**Age**	66	72	72	67	46	67	71	67	53	82	47
**Pathologic stage**	pT1N0 (IA)	pT3N1 (IIIA)	pT2N1 (IIB)	pT2N0 (IIA)	ypT1N1mi (IB)	T2N1 (IIB)	pT1N3 (IIIC)	pT2N0 (IIA)	pT2N0 (IIA)	pT2N2 (IIIA)	T2N0 (IIA)
**Molecular subtype**	Luminal A	Luminal A	Luminal B	HER2-enriched	HER2-enriched	HER2-enriched	Basal-like	Basal-like	Basal-like	Basal-like	Basal-like
**Immunohistochemistry**											
ER	positive	positive	positive	negative	negative	negative (weak positive)	negative	negative	negative	negative	negative
PR	negative	positive	positive	negative	negative	negative (weak positive)	negative	negative	negative	negative	negative
HER2	2+/3	1+/3	3+/3	3+/3	3+/3	3+/3	1+/3	2+/3	1+/3	1+/3	1+/3
HER2 FISH	negative	-	-	-	-	-	-	negative	-	-	-
**No. of single cells**											
*Tumor	26 (22)	56 (53)	37 (33)	59 (55)	77 (76)	25 (18)	51 (50)	23 (22)	60 (55)	16 (15)	11 (11)
Lymph node	-	-	55 (53)	-	-	-	53 (52)	-	-	-	-
**Lymph node metastasis**	0/18	1/13	2/18	0/11	1/4	1/18	16/35	0/3	0/3	9/21	0/6

ER, estrogen receptor; IDC, invasive ductal carcinoma; HER2, human epidermal growth factor receptor 2; PR, progesterone receptor; FISH, fluorescence in situ hybridization

* the number of total cells (the number of analysed cells)

Supplementary Table 2. Tumor-specific gene lists identified at a single cell resolution

a. ER+ tumor marker gene

Gene symbol	Fold change	P-value (LRT test)	AUC (ROC test)
CPB1	11.76	0.0.E+00	0.81
RP11-53O19.1	8.51	0.0.E+00	0.82
TFF1	7.82	0.0.E+00	0.73
PVALB	7.79	0.0.E+00	0.81
ANKRD30B	6.86	0.0.E+00	0.78
LINC00173	6.83	0.0.E+00	0.73
DSCAM-AS1	6.70	0.0.E+00	0.70
IGHG1	6.26	4.9.E-14	0.76
SERPINA5	6.23	0.0.E+00	0.75
ESR1	5.60	0.0.E+00	0.94
LRP2	5.30	0.0.E+00	0.80
IGLC3	5.24	5.9.E-13	0.72
CA12	5.23	0.0.E+00	0.96
RP11-64B16.2	5.23	0.0.E+00	0.72
SLC7A2	5.01	0.0.E+00	0.79
AFF3	4.83	0.0.E+00	0.85
IGFBP4	4.13	0.0.E+00	0.76
GSTM3	3.97	0.0.E+00	0.83
ANKRD30A	3.85	0.0.E+00	0.73
GSTT1	3.82	3.3.E-16	0.71
GSTM1	3.78	3.6.E-14	0.71
AC026806.2	3.66	0.0.E+00	0.74
C19orf33	3.55	0.0.E+00	0.91
STC2	3.53	1.1.E-14	0.75
HSPB8	3.45	0.0.E+00	0.92
RPL29P11	3.42	0.0.E+00	0.74
FBP1	3.36	0.0.E+00	0.78
AGR3	3.25	0.0.E+00	0.85
TCEAL1	3.23	0.0.E+00	0.83
CYP4B1	3.18	4.9.E-12	0.71
SYT1	3.13	2.1.E-12	0.70
COX6C	3.04	0.0.E+00	0.95
MT1E	3.01	0.0.E+00	0.88
SYTL2	3.00	3.6.E-15	0.76
THSD4	2.94	2.1.E-15	0.72
IFI6	2.86	3.5.E-12	0.72
KIAA1467	2.81	1.1.E-16	0.75
SLC39A6	2.76	0.0.E+00	0.86
ABCD3	2.74	1.3.E-13	0.74
SERPINA3	2.71	0.0.E+00	0.82
DEGS2	2.70	1.1.E-16	0.78
ERLIN2	2.69	1.5.E-10	0.73
HEBP1	2.69	1.8.E-12	0.71
BCL2	2.50	5.1.E-15	0.74
TCEAL3	2.47	5.8.E-15	0.78
PPT1	2.45	0.0.E+00	0.85
SLC7A8	2.43	0.0.E+00	0.82
RP11-96D1.10	2.41	3.9.E-12	0.74
HIST1H4H	2.41	6.7.E-12	0.74
PI15	2.41	0.0.E+00	0.77
PPAPDC1B	2.37	1.7.E-13	0.72
RARRES3	2.37	1.8.E-09	0.71
GALNT6	2.27	1.6.E-13	0.79
IL6ST	2.23	0.0.E+00	0.82
MYC	2.21	5.3.E-12	0.76
BST2	2.20	1.1.E-16	0.76
RP11-658F2.8	2.19	9.7.E-09	0.71
MRPS30	2.17	1.5.E-13	0.76
MAPT	2.17	1.1.E-11	0.70
AMFR	2.16	4.8.E-14	0.82
TCEAL4	2.14	0.0.E+00	0.80
MED13L	2.12	2.1.E-15	0.81
ISG15	2.07	1.2.E-13	0.79
NDUFC2	2.05	0.0.E+00	0.80
TIMP3	2.03	2.2.E-11	0.73
RP13-39P12.3	2.02	2.9.E-12	0.74
PARD6B	2.01	0.0.E+00	0.84

b. HER2+ tumor marker genes

Gene symbol	Fold change	P-value (LRT test)	AUC (ROC test)
MUC19	8.80	0.0.E+00	0.71
RPS12P26	8.18	0.0.E+00	0.78
CEACAM5	7.67	0.0.E+00	0.79
UBD	7.58	0.0.E+00	0.83
H19	6.97	0.0.E+00	0.80
NDST4	6.86	0.0.E+00	0.76
KRT20*	6.70	0.0.E+00	0.71
RP11-483P21.2	6.64	0.0.E+00	0.75
BPIFB2	6.62	0.0.E+00	0.76
EREG	5.95	0.0.E+00	0.76
RP11-697E2.7	4.78	0.0.E+00	0.78
IL8	4.67	0.0.E+00	0.79
ZNF710	4.39	0.0.E+00	0.81
MTND2P28	4.37	0.0.E+00	0.74
RP11-617F23.1	4.28	0.0.E+00	0.79
TFPI	4.26	0.0.E+00	0.79
KRT86	4.20	0.0.E+00	0.72
NR1D1*	3.96	0.0.E+00	0.77
NGRN	3.80	0.0.E+00	0.82
CXCL1	3.76	0.0.E+00	0.76
GRB7*	3.61	0.0.E+00	0.95
IGF2BP2	3.23	1.8.E-13	0.72
SEMA4B	3.16	0.0.E+00	0.75
CIB1	3.08	0.0.E+00	0.74
MIEN1*	3.06	0.0.E+00	0.95
CDC6*	2.95	0.0.E+00	0.84
SIX4	2.93	0.0.E+00	0.81
PTPN13	2.93	2.2.E-16	0.76
MSL1*	2.90	0.0.E+00	0.96
CEACAM6	2.86	5.0.E-13	0.77
CAPS	2.60	6.2.E-14	0.74
PPP1R1B*	2.56	8.5.E-14	0.74
AREG	2.54	0.0.E+00	0.79
ERBB2*	2.48	0.0.E+00	0.95
PEG10	2.47	0.0.E+00	0.80
MED1*	2.43	7.8.E-16	0.77
AP3S2	2.41	1.5.E-10	0.74
IDH2	2.32	3.5.E-14	0.74
RP11-889L3.1	2.32	0.0.E+00	0.83
RPL19*	2.30	0.0.E+00	0.79
CDK12*	2.28	0.0.E+00	0.83
RP11-690G19.3*	2.26	7.4.E-14	0.74
NLK	2.18	0.0.E+00	0.77
CASC3*	2.13	0.0.E+00	0.87
STARD3*	2.12	6.3.E-15	0.70
MED31*	2.09	1.7.E-09	0.76
TXNDC17*	2.08	0.0.E+00	0.78
KPNA2*	2.01	1.4.E-11	0.78

* Genes located in HER2 amplification region (17q12-q21)

c. TNBC tumor marker genes

Gene symbol	Fold change	P-value (LRT test)	AUC (ROC test)
FABP7	9.57	0.0.E+00	0.82
TSPAN8	8.13	0.0.E+00	0.78
CYP4Z1	7.44	0.0.E+00	0.74
HOXA10	7.28	0.0.E+00	0.83
CLDN1	7.11	0.0.E+00	0.71
TMSB15A	6.78	0.0.E+00	0.70
C10orf10	5.67	0.0.E+00	0.80
TRPV6	5.65	0.0.E+00	0.75
HOXA9	5.63	0.0.E+00	0.71
ATP13A4	5.57	0.0.E+00	0.72
GLYATL2	5.12	2.1.E-15	0.74
RP11-48O20.4	5.09	0.0.E+00	0.74
DYRK3	5.05	0.0.E+00	0.70
MUCL1	4.75	0.0.E+00	0.72
ID4	4.74	0.0.E+00	0.83
FGFR2	4.55	0.0.E+00	0.70
SHOX2	4.38	0.0.E+00	0.71
Z83851.1	4.29	3.3.E-16	0.72
CD82	4.14	1.1.E-16	0.78
COL6A1	3.89	2.2.E-16	0.70
KRT23	3.87	0.0.E+00	0.85
GCHFR	3.79	1.4.E-12	0.71
PRICKLE1	3.67	3.3.E-16	0.71
GCNT2	3.65	1.1.E-15	0.74
KHDRBS3	3.57	3.3.E-14	0.71
SIPA1L2	3.55	4.4.E-14	0.75
LMO4	3.53	0.0.E+00	0.80
TFAP2B	3.49	0.0.E+00	0.74
SLC43A3	3.44	1.0.E-11	0.71
FURIN	3.40	0.0.E+00	0.77
ELF5	3.40	1.9.E-13	0.74
C1orf116	3.19	3.7.E-10	0.71
ADD3	3.14	2.2.E-16	0.76
EFNA3	3.12	5.9.E-15	0.71
EFCAB4A	3.11	3.7.E-14	0.74
LTF	3.07	3.6.E-14	0.73
LRRC31	3.02	2.2.E-14	0.71
ARL4C	2.95	1.2.E-13	0.75
GPNMB	2.93	2.0.E-13	0.75
VIM	2.82	3.7.E-12	0.76
SDR16C5	2.81	6.3.E-12	0.72
RHOV	2.80	1.0.E-10	0.71
PXDC1	2.77	1.7.E-10	0.70
MALL	2.75	0.0.E+00	0.74
YAP1	2.74	1.4.E-09	0.71
A2ML1	2.74	1.0.E-15	0.70
RP1-257A7.5	2.72	1.8.E-12	0.71
RP11-353N4.6	2.71	1.3.E-10	0.71
ZBTB18	2.66	0.0.E+00	0.76
CTD-2314B22.3	2.65	2.1.E-10	0.74
GALNT3	2.65	8.6.E-09	0.71
BCL11A	2.57	6.1.E-13	0.70
CXADR	2.55	1.1.E-16	0.77
SSFA2	2.51	7.1.E-13	0.75
ADM	2.46	3.6.E-13	0.73
GUCY1A3	2.43	7.3.E-10	0.72
GSTP1	2.39	0.0.E+00	0.85
ADCK3	2.37	5.7.E-10	0.70
SLC25A37	2.32	0.0.E+00	0.82
SFRP1	2.24	1.7.E-14	0.71
PRNP	2.21	1.2.E-13	0.78
DEGS1	2.18	7.8.E-13	0.76
RP11-110G21.2	2.14	4.6.E-15	0.74
RBMS1	2.14	4.2.E-09	0.72
RP11-640M9.1	2.12	1.8.E-12	0.77
AL589743.1	2.10	2.6.E-13	0.77
ATF3	2.09	2.7.E-12	0.74
SIVA1	2.09	4.3.E-11	0.71
TACSTD2	2.04	1.1.E-16	0.81
HEBP2	2.03	0.0.E+00	0.86

Supplementary Table 3. Immune gene sets

Gene set name	B cells	T cells	T helper cells	CD8 T cells	Cytotoxic cells	NK cells	Dendritic cells	Eosinophils	Macrophages	Mast cells	Neutrophils
Genes	MS4A1	PRKCQ	ICOS	CD8B	KLRD1	LOC643313	CD209	IL5RA	MARCO	PRG2	CSF3R
	TCL1A	CD3D	LRBA	CD8A	KLRF1	GAGE2	CCL17	KCNH2	CXCL5	CTSG	CYP4F3
	HLA-DOB	CD3G	ITM2A	PF4	GNLY	ZNF747	HSD11B1	TKTL1	SCG5	TPSAB1	VNN3
	PNOC	CD28	FAM111A	PRR5	CTSW	XCL1	CCL13	EMR1	SULT1C2	SLC18A2	FPRL1
	KIAA0125	LCK	PHF10	SF1	KLRB1	XCL2	CCL22	CCR3	MSR1	MS4A2	KCNJ15
	CD19	TRAT1	NUP107	LIME1	KLRK1	AF107846	PPFIBP2	ACACB	CTSK	CPA3	MME
	CR2	BCL11B	SEC24C	DNAJB1	NKG7	SLC30A5	NPR1	THBS1	PTGDS	TPSB2	IL8RA
	IGHG1	CD2	NAP1L4	ARHGAP8	GZMH	SGMS1	CD1B	GALC	COLEC12	TPSB2	IL8RB
	FCRL2	TRBC1	BATF	GZMM	SIGIRR	MCM3AP	VASH1	RNU2	GPC4	GATA2	FCGR3B
	BLK	TRAC	ASF1A	SLC16A7	ZBTB16	TBXA2R	F13A1	CLC	PCOLCE2	HDC	DYSF
	COCH	ITM2A	FRYL	SFRS7	RUNX3	CDC5L	CD1E	HIST1H1C	CHIT1	LOH11CR2A	FCAR
	OSBPL10	SH2D1A	FUSIP1	APBA2	APOL3	LOC730096	MMP12	CYSLTR2	KAL1	SIGLEC6	CEACAM3
	IGHA1	CD6	YME1L1	C4orf15	RORA	FUT5	FABP4	HRH4	CLEC5A	ELA2	HIST1H2BC
	TNFRSF17	CD96	TRA	LEPROTL1	APBA2	FGF18	CLEC10A	RNASE2	ME1	CMA1	HPSE
	ABCB4	NCALD	RPA1	ZFP36L2	WHDC1L1	MRC2	SYT17	CAT	DNASE2B	PGDS	CPPED1
	BLNK	GIMAP5	UBE2L3	GADD45A	DUSP2	RP5-886K2.1	MS4A6A	LRP5L	CCL7	MLPH	CREB5
	GLDC	TRA	ANP32B	MYST3	GZMA	SPN	CTNS	SYNJ1	FN1	ADCYAP1	S100A12
	MEF2C	CD3E	DDX50	ZEB1		PSMD4	GUCA1A	THBS4	CD163	SLC24A3	TNFRSF10C
	IGHM	SKAP1	C13orf34	ZNF609		PRX	CARD9	GPR44	GM2A	CALB2	SLC22A4
	FAM30A		PPP2R5C	C12orf47		FZR1	ABCG2	KBTBD11	SCARB2	KIT	TECPR2
	SPIB		SLC25A12	THUMPD1		ZNF205	CD1A	HES1	BCAT1	TAL1	SLC25A37
	BCL11A		ATF2	VAMP2		AL080130	PPARG	ABHD2	RAI14	ABCC4	BST1
	GNG7		CD28	ZNF91		ZNF528	RAP1GAP	TIPARP	COL8A2	PPM1H	CRISPLD2
	IGKC		GOLGA8A	ZNF22		MAPRE3	SLC7A8	SMPD3	APOE	MAOB	G0S2
	CD72		IFNG	TMC6		BCL2	GSTT1	MYO15B	CHI3L1	HPGD	SIGLEC5
	MICAL3		LTA	FLT3LG		NM_017616	PDXK	TGIF1	ATG7	SCG2	CD93
	BACH2		APBB2	CDKN2AIP		ARL6IP2	FZD2	RRP12	CD84	PTGS1	MGAM
	IGL		DOK5	TSC22D3		PDLIM4	CSF1R	IGSF2	FDX1	CEACAM8	ALPL
	CCR9		IL12RB2	TBCC		TRPV6	HS3ST2	RCOR3	MS4A4A	MPO	FPR1
	QRSL1		APOD	RBM3		LDB3	CH25H	EPN2	SGMS1	NR0B1	PDE4B
	DTNB		ZBTB32	ABT1		ADARB1	LMAN2L	C9orf156	EMP1	LOC339524	LILRB2
	HLA-DQA1		CD38	C19orf6		SMEK1	SLC26A6	SIAH1	CYBB		
	SCN3A		CSF2	CAMLG		TCTN2	BLVRB		CD68		
	SLC15A2		CTLA4	PPP1R2		TINAGL1	NUDT9				
			CD70	AES		IGFBP5	PREP				
			DPP4	KLF9		ALDH1B1	TM7SF4				
			EGFL6	PRF1		NCR1	TACSTD2				
			BST2			KIR3DL2	CD1C				
			DUSP5			SPON2	CCL1				
			LRP8			KIR2DL3	EBI3				
			IL22			GZMB	INDO				
			DGKI			KIR3DS1	LAMP3				
			CCL4			KIR3DL1	OAS3				
			GGT1			TCC38	IL3RA				
			LRRN3			PMEPA1					
			SYNGR3			IL21R					
			ATP9A			KIR3DL3					
			BTG3			KIR2DS5					
			CMAH			KIR2DS2					
			HBEGF			GTF3C1					
			SGCB			KIR2DS1					
			PMCH			S1PR5					
			AHI1			DUSP4					
			PTGIS			RRAD					
			CXCR6			PLA2G6					
			EVI5			NIBP					
			IL26			FOXJ1					
			MB			MARCH6					
			NEIL3			MADD					
			GSTA4			LPCAT4					
			PHEX			MPPED1					
			SMAD2			MUC3B					
			CENPF								
			ANK1								
			ADCY1								
			LOC728210								
			LAIR2								
			SNRPD1								
			MICAL2								
			DHFR								
			WDHD1								
			BIRC5								
			SLC39A14								
			HELLS								
			LIMA1								
			CDC25C								
			CDC7								
			GATA3								

Supplementary Table 4. Immune-specific gene lists identified at a single cell resolution

a. B cell upregulated

Gene symbol	Fold change	P-value (LRT test)	AUC (ROC test)	Gene symbol	Fold change	P-value (LRT test)	AUC (ROC test)	Gene symbol	Fold change	P-value (LRT test)	AUC (ROC test)
IGLC1	12.37	3.9.E-12	0.70	DCAF12	4.43	1.6.E-11	0.71	SEL1L3	2.69	4.9.E-11	0.79
FCRLA	8.11	0.0.E+00	0.84	ABCA6	4.26	1.8.E-11	0.72	BCAR3	2.67	4.9.E-10	0.73
RN7SL627P	7.98	3.7.E-15	0.74	GPR18	4.16	8.9.E-16	0.81	SSBP2	2.66	3.3.E-12	0.75
RN7SL639P	7.98	3.7.E-15	0.74	PAX5	4.11	0.0.E+00	0.89	BCAS4	2.66	4.2.E-15	0.77
GCSAM	7.81	7.8.E-16	0.75	CTA-250D10.23	4.09	0.0.E+00	0.77	FAM3C	2.65	2.0.E-08	0.72
VNN2	7.78	0.0.E+00	0.77	MCTP2	4.06	1.6.E-12	0.71	TMED8	2.65	2.7.E-11	0.75
AICDA	7.77	0.0.E+00	0.78	CDK14	4.04	7.7.E-12	0.74	FAM210A	2.64	3.0.E-08	0.70
IGHG4	7.68	0.0.E+00	0.80	BRI3BP	4.01	2.5.E-11	0.72	KIAA0922	2.64	2.1.E-12	0.80
VPREB3	7.17	0.0.E+00	0.84	LPP	3.93	5.1.E-14	0.76	PTK2	2.60	3.4.E-08	0.73
RGS13	7.16	0.0.E+00	0.85	NCF1	3.84	1.7.E-12	0.71	MZB1	2.60	5.5.E-08	0.71
SNX29P1	7.05	0.0.E+00	0.80	ENTPD4	3.83	6.1.E-10	0.72	KBTBD8	2.55	1.8.E-13	0.78
E2F5	6.77	1.8.E-13	0.70	DTX1	3.81	7.9.E-13	0.72	FAM208B	2.49	1.7.E-08	0.73
PNOC	6.68	7.2.E-14	0.72	POU2AF1	3.75	0.0.E+00	0.82	LAT2	2.46	4.0.E-11	0.79
FCRL1	6.65	2.4.E-14	0.72	RRM2B	3.70	2.1.E-10	0.72	BIK	2.40	9.7.E-14	0.77
AC079767.4	6.55	2.2.E-12	0.70	RAB30	3.70	0.0.E+00	0.87	HLA-DOB	2.37	6.9.E-09	0.72
ELL3	6.48	0.0.E+00	0.78	FAM3C2	3.51	1.7.E-11	0.70	ADAM28	2.35	7.0.E-10	0.76
IGHG1	6.37	0.0.E+00	0.86	RRAS2	3.51	4.3.E-14	0.79	SEC14L1	2.34	1.4.E-07	0.75
EBF1	6.11	0.0.E+00	0.82	SHCBP1	3.50	4.0.E-10	0.72	PHF6	2.33	3.3.E-08	0.73
CCDC144B	6.09	0.0.E+00	0.80	CTD-2369P2.2	3.44	1.7.E-14	0.82	MEF2C	2.33	0.0.E+00	0.88
IGKC	6.09	5.6.E-16	0.85	DNAJC10	3.39	1.6.E-12	0.77	UBE2J1	2.31	4.0.E-09	0.76
CD79A	6.02	0.0.E+00	0.92	PRKD3	3.35	2.5.E-14	0.82	CLIC4	2.29	3.0.E-06	0.71
CD19	5.89	0.0.E+00	0.78	HAUS8	3.23	1.0.E-07	0.71	TRAK1	2.25	7.9.E-07	0.70
LINC00877	5.87	2.1.E-13	0.71	KLHL6	3.22	2.6.E-10	0.75	RPRD1B	2.22	2.0.E-08	0.72
CCDC144A	5.86	4.4.E-16	0.76	LRMP	3.10	0.0.E+00	0.86	SWAP70	2.12	0.0.E+00	0.87
STAG3	5.85	1.6.E-11	0.71	HDAC9	3.10	7.8.E-16	0.82	LY9	2.12	1.9.E-10	0.75
RALGPS2	5.80	3.3.E-16	0.76	P2RX5	3.09	6.3.E-12	0.72	FAM76B	2.08	2.3.E-09	0.74
ZNF608	5.64	0.0.E+00	0.78	COBLL1	3.06	7.5.E-15	0.79	PIK3C2B	2.07	1.0.E-10	0.71
CD22	5.57	0.0.E+00	0.89	ST6GAL1	3.04	0.0.E+00	0.84	RHOH	2.04	2.1.E-12	0.81
PRPSAP2	5.40	5.6.E-16	0.80	DGKD	2.97	6.1.E-09	0.72	CCNB1	2.04	1.0.E-06	0.72
MS4A1	5.39	0.0.E+00	0.97	BLNK	2.91	6.4.E-12	0.79	MRPS27	2.03	2.6.E-08	0.72
P2RY8	5.33	1.1.E-13	0.71	BANK1	2.81	5.2.E-13	0.74	SPATS2	2.03	2.5.E-07	0.71
IGHG3	5.11	0.0.E+00	0.86	SMIM14	2.79	0.0.E+00	0.81	SNX22	2.02	2.8.E-12	0.72
GMDS	5.06	2.3.E-11	0.71	EIF2AK3	2.75	2.7.E-13	0.79	TMEM156	2.02	4.9.E-07	0.72
NEIL1	4.96	1.1.E-12	0.74	EAF2	2.74	0.0.E+00	0.85	IGLC3	2.01	1.9.E-08	0.71
SNX29P2	4.93	0.0.E+00	0.91	LYPLAL1	2.73	1.6.E-07	0.71	NR4A1	2.00	6.6.E-06	0.71
PLCG2	4.85	0.0.E+00	0.83	TEX9	2.72	3.2.E-12	0.71				
CD79B	4.55	0.0.E+00	0.89	FANCA	2.72	2.3.E-09	0.71				

b. T cell upregulated

Gene symbol	Fold change	P-value (LRT test)	AUC (ROC test)	Gene symbol	Fold change	P-value (LRT test)	AUC (ROC test)	Gene symbol	Fold change	P-value (LRT test)	AUC (ROC test)
IFNG	6.19	7.7.E-10	0.71	RORA	3.63	3.7.E-10	0.75	CD96	2.78	6.0.E-14	0.81
CD3G	5.06	0.0.E+00	0.90	SH2D1A	3.43	1.1.E-16	0.79	MAF	2.77	1.0.E-07	0.73
CD3D	4.46	0.0.E+00	0.94	PRKCH	3.30	1.0.E-09	0.75	CCL5	2.75	3.8.E-06	0.72
INPP4B	4.07	3.0.E-11	0.72	TRAT1	3.24	5.0.E-10	0.71	TIGIT	2.73	1.1.E-09	0.75
CD2	4.06	0.0.E+00	0.95	FYN	3.07	2.7.E-13	0.82	SLA	2.68	6.8.E-09	0.74
ITK	3.90	8.9.E-10	0.73	ARAP2	2.93	5.3.E-09	0.75	TRAC	2.66	2.2.E-16	0.87
STAT4	3.77	9.6.E-12	0.77	ITM2A	2.82	1.1.E-16	0.82	TRBC2	2.35	0.0.E+00	0.91
IL32	3.64	0.0.E+00	0.91	CD3E	2.81	2.2.E-15	0.78	RARRES3	2.01	3.2.E-06	0.73

Table continued on next page

Supplementary Table 4 cont.

c. Macrophage upregulated

Gene symbol	Fold change	P-value (LRT test)	AUC (ROC test)	Gene symbol	Fold change	P-value (LRT test)	AUC (ROC test)	Gene symbol	Fold change	P-value (LRT test)	AUC (ROC test)
C1QB	9.79	2.3.E-12	0.72	PLXDC2	3.73	4.6.E-11	0.74	CST3	2.64	0.0.E+00	0.93
CD163	9.28	1.1.E-15	0.73	CREG1	3.70	3.8.E-11	0.75	VAMP3	2.62	9.5.E-09	0.74
IL1B	9.14	2.8.E-14	0.72	MNDA	3.69	8.1.E-13	0.81	VAMP5	2.61	1.6.E-06	0.74
FCGR3A	8.61	4.5.E-14	0.75	KIAA1598	3.68	2.1.E-08	0.73	BEST1	2.60	6.4.E-09	0.79
CCL2	8.52	1.9.E-11	0.72	GPNMB	3.67	7.3.E-09	0.72	RBM47	2.58	2.1.E-07	0.76
TMEM176B	8.24	0.0.E+00	0.80	MYOF	3.66	9.3.E-10	0.77	CEBPB	2.52	2.0.E-07	0.70
CXCL9	7.97	7.3.E-12	0.73	TIMP1	3.65	2.0.E-07	0.75	PSAP	2.51	1.7.E-09	0.82
FCGR1A	7.69	1.4.E-11	0.71	AXL	3.65	3.6.E-15	0.80	TGFBI	2.50	6.9.E-14	0.83
APOC1	7.59	5.2.E-15	0.79	LAIR1	3.60	1.4.E-10	0.75	CXCL16	2.49	1.0.E-08	0.73
FN1	7.51	3.3.E-16	0.83	CD68	3.60	1.6.E-14	0.85	TIMP2	2.48	2.3.E-12	0.78
TMEM176A	7.09	3.9.E-12	0.72	MS4A6A	3.60	1.1.E-16	0.84	ITM2B	2.47	2.2.E-08	0.80
S100A9	6.72	2.1.E-10	0.73	TYROBP	3.55	0.0.E+00	0.88	CPVL	2.44	2.2.E-07	0.70
APOE	6.68	1.4.E-12	0.71	RASSF4	3.54	3.0.E-11	0.77	IGSF6	2.44	9.8.E-12	0.75
SLAMF8	6.66	2.9.E-11	0.71	DSE	3.51	3.3.E-16	0.78	HEXB	2.42	1.5.E-09	0.79
CD14	6.35	4.2.E-13	0.76	FTL	3.50	0.0.E+00	0.88	CTSC	2.41	8.6.E-10	0.81
CXCL10	6.05	5.4.E-10	0.75	KCTD12	3.23	1.2.E-13	0.77	BRI3	2.39	1.9.E-07	0.73
FCGR2A	5.72	7.2.E-12	0.75	FCER1G	3.21	0.0.E+00	0.87	GPX1	2.36	7.1.E-11	0.84
RP11-1143G9.4	5.63	1.3.E-15	0.81	FCGRT	3.21	5.3.E-11	0.79	MARCKS	2.36	1.6.E-07	0.77
HNMT	5.55	4.4.E-12	0.71	CTSB	3.17	3.3.E-13	0.85	CAPG	2.33	7.8.E-08	0.79
PLBD1	5.48	7.3.E-14	0.70	HLA-DQB2	3.09	5.4.E-05	0.72	IFITM3	2.33	1.1.E-10	0.83
A2M	5.35	0.0.E+00	0.82	SLC8A1	3.05	2.8.E-07	0.70	S100A11	2.31	1.8.E-11	0.88
CXCL2	5.06	1.8.E-10	0.74	IER3	3.04	4.2.E-08	0.72	HLA-DQA2	2.29	1.3.E-07	0.77
MAFB	5.02	1.1.E-16	0.80	MFSD1	3.03	2.7.E-11	0.82	LST1	2.28	6.4.E-10	0.80
MSR1	4.87	1.8.E-12	0.75	PLAUR	3.00	5.4.E-13	0.81	TNFSF13B	2.25	4.4.E-11	0.80
PLAU	4.81	1.6.E-09	0.72	IL18	3.00	2.8.E-10	0.74	RNASET2	2.23	1.7.E-07	0.77
CTSL	4.69	3.1.E-14	0.82	ANKRD22	2.96	1.5.E-09	0.72	FTLP3	2.23	2.4.E-10	0.80
SIRPA	4.66	1.9.E-13	0.76	ATP6AP1	2.93	9.2.E-07	0.71	LPCAT2	2.20	2.6.E-11	0.71
FGL2	4.62	6.2.E-12	0.76	SOD2	2.90	9.7.E-08	0.77	CD63	2.20	1.8.E-07	0.78
LYZ	4.55	2.3.E-15	0.82	GLUL	2.86	8.5.E-09	0.79	CCL4	2.20	2.7.E-04	0.70
SERPING1	4.51	0.0.E+00	0.88	IFIT1	2.83	4.4.E-06	0.71	LILRB4	2.19	1.5.E-09	0.75
SCARB2	4.40	4.2.E-11	0.76	IFI27	2.82	1.5.E-13	0.87	HLA-DRB6	2.16	6.2.E-08	0.72
CLEC7A	4.36	7.9.E-15	0.84	CCND1	2.79	7.4.E-08	0.71	MS4A7	2.13	2.2.E-06	0.72
AIF1	4.20	6.1.E-14	0.83	IFIT3	2.71	4.5.E-05	0.71	ATOX1	2.12	3.0.E-06	0.75
PILRA	4.19	6.1.E-11	0.71	GNAQ	2.71	4.1.E-11	0.77	DAB2	2.09	2.2.E-15	0.81
C15orf48	4.00	4.0.E-12	0.76	IL8	2.71	8.8.E-08	0.76	NAGK	2.02	5.2.E-07	0.76
FAM26F	3.97	3.0.E-09	0.71	NPC2	2.70	1.0.E-15	0.90	IFI30	2.02	6.6.E-09	0.81
SERPINA1	3.80	9.7.E-11	0.75	NRP1	2.70	1.7.E-08	0.73				
RIN2	3.77	5.6.E-10	0.73	RNASE6	2.66	4.3.E-07	0.73				

Supplementary Table 5. Immune signature gene sets

a. M1/M2 gene sets

Gene set name	M1_UP	M2_UP
Genes	CCR7	GPR86
	IL2RA	P2RY5
	IL15RA	TGFBR2
	IL7R	HRH1
	CXCL11	TLR5
	CCL19	DCL-1
	CXCL10	MSR1
	CXCL9	CXCR4
	TNF	DECTIN1
	CCL5	P2RY14
	CCL15	DCSIGN
	IL12B	CLECSF13
	IL15	MS4A6A
	TRAIL	CD36
	IL6	MS4A4A
	CCL20	MRC1
	PBEF1	IGF1
	ECGF1	CCL23
	BCL2A1	CCL18
	FAS	CCL13
	BIRC3	SLC21A9
	GADD45G	SLC4A7
	HSXIAPAF1	SLC38A6
	SLC7A5	CTSC
	SLC21A15	HEXB
	SLC2A6	LIPA
	SLC31A2	ADK
	INDO	HNMT
	PLA1A	TPST2
	OASL	CERK
	CHI3L2	HS3ST2
	HSD11B1	LTA4H
	AK3	CA2
	SPHK1	ALOX15
	PFKFB3	HS3ST1
	PSME2	TGFBI
	PFKP	SEPP1
	PSMB9	CHN2
	PSMA2	FN1
	OAS2	FGL2
	PTX3	GAS7
	CSPG2	EGR2
	APOL3	MAF
	IGFBP4	
	APOL1	
	PDGFA	
	EDN1	
	APOL2	
	INHBA	
	APOL6	
	HESX1	
	IRF1	
	ATF3	
	IRF7	

b. B cell signature gene sets

Gene set name	Anti-apoptosis	Naïve / Memory	Cytokines	Pro-apoptosis	Proliferation	Germinal center
Genes	BCL2	BMI1	CCL5	BIK	ASK	BCL7A
	CASP8	CD24	CCR1	FAS	AURKA	CD27
	FAIM3	CD69	CCR6	LGALS1	AURKB	CD36
	TNFSF10	CR1	CCR7		AURKC	CD80
		ENTPD1	CLR1		BUB1B	HGF
		FCER2	GPR9		CCNA2	MME
		FCGR2B	IFNGR1		CCNB1	MYBL1
		FCGRT	IL10RB		CCNB2	RGS13
		IGHD	IL15		CCNE2	TNFSF8
			IL24		CCNF	
			IL2RB		CDC20	
			IL2RG		CDC25B	
			IL4R		CDC45	
			IL6		CDC6	
			IL8		CDK1	
			TGFB1		CDK5	
			TGFB3		CDKN2C	
			TGFBR2		CENPA	
			TNFRSF1B		CENPE	
			TNFSF11		CENPF	
			XCL1		CHEK1	
					CIP2	
					DEEPEST	
					E2F5	
					ECA39	
					FOXA1	
					GADD45A	
					GADD45B	
					GTSE1	
					HEC1	
					KIF11	
					KIF22	
					KIF23	
					MAD2L1	
					MCM3	
					MCM4	
					MCM6	
					MCM7	
					MKI67	
					NCAPD2	
					NDN	
					NEK2	
					PCNA	
					PLK4	
					PTTG1	
					RABGAP1	
					RAD17	
					RFC3	
					RGL2	
					RPA3	
					SIL-TAL1	
					TFDP1	
					TMPO	
					TPX2	
					TTK	
					UBE2C	
					WEE1	
					ZW10	

Table continued on next page

Supplementary Table 5 cont.

c. T cell signature gene sets

Gene set name	Cytotoxic	Exhausted	Regulatory	Naïve	Costimulatory	G1/S	G2/M
Genes	CST7	BTLA	IL2RA	CCR7	ICOS	ATAD2	ANLN
	GZMA	CTLA4	IL4R	LEF1	CD226	BLM	ANP32E
	GZMB	HAVCR2	IL7	SELL	SLAMF1	BRIP1	AURKA
	IFNG	LAG3	TGFB1	TCF7	TNFRSF14	CASP8AP2	AURKB
	NKG7	PDCD1	TGFB3		TNFRSF25	CCNE2	BIRC5
	PRF1	TIGIT	TGFBI		TNFRSF9	CDC45	BUB1
	TNFSF10		TGFBR1			CDC6	CBX5
						CDCA7	CCNB2
						CHAF1B	CDC20
						CLSPN	CDC25C
						DSCC1	CDCA2
						DTL	CDCA3
						E2F8	CDCA8
						EXO1	CDK1
						FEN1	CENPA
						GINS2	CENPE
						GMNN	CENPF
						HELLS	CKAP2
						MCM2	CKAP2L
						MCM4	CKAP5
						MCM5	CKS1B
						MCM6	CKS2
						MLF1IP	CTCF
						MSH2	DLGAP5
						NASP	ECT2
						PCNA	FAM64A
						POLA1	G2E3
						POLD3	GAS2L3
						PRIM1	GTSE1
						RAD51	HJURP
						RAD51AP1	HJURP
						RFC2	HMGB2
						RPA2	HMMR
						RRM1	HN1
						RRM2	KIF11
						SLBP	KIF20B
						TIPIN	KIF23
						TYMS	KIF2C
						UBR7	LBR
						UHRF1	MKI67
						UNG	NCAPD2
						USP1	NDC80
						WDR76	NEK2
							NUF2
							NUSAP1
							PSRC1
							RANGAP1
							SMC4
							TACC3
							TMPO
							TOP2A
							TPX2
							TTK
							TUBB4B
							UBE2C

Supplementary Table 6. Quantitative PCR primers

RefSeq ID	Gene Symbol	Forward Primer	Reverse Primer
NM_002046	GAPDH	AGGTCGGAGTCAACGGATTT	TGACGGTGCCATGGAATTTG
NM_001101	ACTB	ACTCTTCCAGCCTTCCTTCC	CGTACAGGTCTTTGCGGATG
NM_000125	ESR1	GACAGGGAGCTGGTTCACA	GACCTGATCATGGAGGGTCAAA
NM_000926	PGR	AGCCAAGAAGAGTTCCTCTGTA	TTGACTTCGTAGCCCTTCCA
NM_004448	ERBB2	ACAGGGAAAGCTGTGGGAAA	TACGCCTCCAACACACTGAA
NM_005228	EGFR	AGGTGAAAACAGCTGCAAGG	CCAGAAGGTTGCACTTGTCC
NM_000633	BCL2	ATGTGTGTGGAGAGCGTCAA	GTGCCGGTTCAGGTACTCA
NM_002417	MKI67	AGAGTAACGCGGAGTGTCA	CTTGACACACACATTGTCCTCA
NM_000044	AR	CTTTGCAGCCTTGCTCTCTA	TCTGGTCGTCCACGTGTAA
NM_000546	TP53	GACTGTACCACCATCCACTACA	AAAGCTGTTCCGTCCCAGTA
NM_001012270	BIRC5	GGACCACCGCATCTCTACAT	GAAACACTGGGCCAAGTCTG
NM_018014	BCL11A	AACCCCAGCACTTAAGCAAAC	GGCCGTGGTCTGGTTCA
NM_000224	KRT18	TCCCATGTCCCAGTCAATTCC	TACCTGGGAGGGGATGTTCA
NM_002276	KRT19	GGAGGTGTCATTGGAGCTGAA	AGCAGCTTCCACCACTTCAA
NM_002051	GATA3	CACGGTGCAGAGGTACCC	AGGGTAGGGATCCATGAAGCA
NM_004360	CDH1	AGTGCCAACTGGACCATTCA	TCTAAGGCCATCTTTGGCTTCA
NM_001067	TOP2A	GGTGTGGAACTAGAAGGCCT	TCTGTTTCTCGTGGAGGGAC
NM_000201	ICAM1	AACCCCACAGTCACCTATGG	TTCTGAGACCTCTGGCTTCG
NM_001432	EREG	TTGTTTGCATGGACAGTGCA	GCTTAAAGGTTGGTGGACGG
NM_080921	PTPRC	GTGGCTTAAACTCTTGGCATTT	GGGAAGGTGTTGGGCTTT
NM_002354	EPCAM	CGTCAATGCCAGTGTACTTCA	TTCTGCCTTCATCACCAAACA
NM_201442.2	C1S	TACGGGGTTTGCTGCATACTA	AGTGGCTACAAGGGACATCTAC
NM_000064.3	C3	GGCGTGTTCGTGCTGAATAA	CCGATGTCTGCCTTCTCCA
NM_000346	SOX9	GTGCTCAAAGGCTACGACTG	AGAAGTCTCCAGAGCTTGCC
